# Gout, in Its Protean Aspects

**Published:** 1883-12

**Authors:** 


**Gout, in its Protean Aspects** By J. MILNER FOTHERGILL, M. D. Detroit, Michigan: George S. Davis, Publisher. 1883.

Whatever comes from the pen of Dr. Fothergill, on digestion and mal-assimilation, commands attention from the profession, as much from the research of which all his writings on medical subjects bear evidence, as from the practical manner with which he treats the subject in hand. The present work of 300 pages is another contribution on a subject which will be sure to interest a large number of professional readers. Gout, in its disguised forms, has puzzled many of our readers, and a solution of the problem, after various and almost futile attempts, has only been made through a recognition of the principles which are elucidated in the work before us.

We commend the book most heartily. Its perusal has given us much satisfaction, and we are sure the profession will appreciate it.

